# Case report of *Actinomyces turicensis* meningitis as a complication of purulent mastoiditis

**DOI:** 10.1186/s12879-018-3610-y

**Published:** 2018-12-20

**Authors:** Béla Kocsis, Zoltán Tiszlavicz, Gabriella Jakab, Réka Brassay, Márton Orbán, Ágnes Sárkány, Dóra Szabó

**Affiliations:** 1 Semmelweis University, Institute of Medical Microbiology, Budapest, Hungary; 2 SYNLAB Székesfehérvár Microbiology Laboratory, Székesfehérvár, Hungary; 3 “Szent György” University Teaching Hospital, Székesfehérvár, Hungary

**Keywords:** *Actinomyces turicensis*, Anaerobic culture, Cerebrospinal fluid, Mastoiditis, Meningitis, Poor socioeconomic condition

## Abstract

**Background:**

Central nervous system (CNS) infections caused by *Actinomyces spp*. including brain abscess, actinomycoma, subdural empyema and epidural abscess are well described, however reports of Actinomyces-associated meningitis are scarcely reported.

**Case report:**

We present the case of a 43-year-old Hungarian male patient with poor socioeconomic status who developed acute bacterial meningitis caused by *Actinomyces turicensis* originating from the left side mastoiditis. The bacterial cultures of both cerebrospinal fluid (CSF) and purulent discharge collected during the mastoid surgery showed slow growing Gram-positive rods that were identified by automated systems (API, VITEK) as *A. turicensis* The bacterial identification was confirmed by 16S rRNA PCR and subsequent nucleic acid sequencing. No bacterial growth was detected in blood culture bottles after 5 days of incubation. Hence, multiple antibacterial treatments and surgical intervention the patient passed away.

**Conclusions:**

Anaerobes are rarely involved in CNS infections therefore anaerobic culture of CSF samples is routinely not performed. However, anaerobic bacteria should be considered as potential pathogens when certain risk factors are present, such as paranasal sinusitis, mastoiditis in patients with poor socioeconomic condition. To the best of our knowledge, our case report is the first description of *A. turicensis* meningitis that has been diagnosed as consequence of purulent mastoiditis.

## Background

Meningitis caused by anaerobic bacteria is a rare disease therefore anaerobic culture of the cerebrospinal fluid (CSF) samples is not generally recommended. However, by these sparse cases of anaerobic meningitis certain conditions present risk factors namely, recurrent or chronic otitis media, brain abscess, necrotizing bowel lesions or peritonitis [1]. In cases of identifiable risk factors anaerobic meningitis should be kept in mind and the CSF specimen should be cultured both aerobically and anaerobically. The routine CSF sample processing lacks the anaerobic incubation that can lead to false-negative results and as a consequence to misdiagnose and therapy failure.

Actinomyces genus contains strictly anaerobic and aerotolerant, non-acid-fast, Gram-positive organisms with variable morphology, ranging from characteristic branching rods to coccobacilli arranged singly or in pairs. The most well-known species is *Actinomyces israelii*, however altogether 25 published Actinomyces species from human samples have been described [2]. Among these species *Actinomyces turicensis* was first reported in 1995 [3] and has been emerging as an important cause of infections [4]. *A. turicensis* is a non-pigment producing, aerotolerant member of Actinomyces genus. Its biochemical characteristics include negative reactions for the production of catalase, urease, β-N-acetyl-glucosaminidase and β-galactosidase, nitrate reduction, esculin hydrolysis, CAMP test and the fermentation of mannitol additionally, it produces α-glucosidase and ferments maltose and sucrose. Variable reactions are raffinose and trehalose fermentation [3].

*Actinomyces spp*. belong to the normal bacterial flora in the oral cavity, pharynx, gut and genitourinary tract [5–10]. Actinomycosis is an uncommon infection caused by *Actinomyces spp*. It is characterized by an indolent, slow growing, granulomatosus disease and categorized based on the anatomical sites as orocervicofacial, thoracic and abdominopelvic forms, or in some other cases it appears as cutaneous or musculoskeletal [11–14]. Actinomyces infections of central nervous system (CNS) such as brain abscess and meningitis are rarely diagnosed [15–18]. To date no published reports of *A. turicensis* meningitis were found on online databases. We herein report the first case of *A. turicensis* meningitis that originated from the left mastoiditis and it was diagnosed in a patient with poor socioeconomic status.

## Case presentation

A 43-year-old Hungarian man with poor socioeconomic living conditions was found lying on the floor unresponsively by a family member. He was taken to a regional hospital “Szent György” University Teaching Hospital, Székesfehérvár, Hungary. On admission he had low level of consciousness accompanied by stiff neck, constricted pupils and fever (38.6 °C). Alcohol abuse, smoking and epileptic seizures were found in his past medical records. Urgent skull CT scan was performed revealing left side mastoiditis but neither brain abscess nor vascular disorders were described. Blood was drawn for clinical chemistry and for bacterial culture. Relevant parameters of the blood test showed elevated white blood cell (WBC) count 24.1 10^9^/L (87.5% Neutrophils), increased C-reactive protein and procalcitonin levels (211.4 mg/L and 0.46 ng/ml, respectively). Laboratory parameters of blood and liquor are shown in Table 1. The results of the urine tests were normal.

**Table 1:** Laboratory parameters of blood and cerebrospinal fluid

Blood results		
White blood cells	24.1 × 10^9^/L	+
Red blood cells	4.7 × 10^12^/L	normal
Hemoglobin	140 g/L	normal
Neutrophil	87.5%	+
Glucose	8.1 mmol/L	+
Total protein	74 g/L	normal
GOT	101 U/L	+
GPT	122 U/L	+
GGT	257 U/L	+
ALP	221 U/L	+
LDH	719 U/L	+
CRP	211.4 mg/L	normal
Procalcitonin	0.46 ng/L	normal
Liquor results		
Liquor glucose	0.6 mmol/L	–
Liquor protein	12.4 g/L	+

Lumbar puncture was carried out and the CSF sample was taken to the laboratory immediately. The slightly xanthochromic CSF was cloudy showing increased WBC count (7400 cells/μl), elevated protein level (12.4 g/L), and low glucose level (< 0.6 mmol/L) compared to the elevated serum glucose level (8.1 mmol/L).

After taking blood and CSF specimens for culture Ceftriaxone (2 × 2 g), Vancomycin (2 × 1 g) and Ampicillin (6 × 2 g) were started and completed with supportive treatment.

The patient’s CSF sample was processed routinely in our Microbiology laboratory (SYNLAB Székesfehérvár, Hungary) on arrival. The Pastorex Meningitis agglutination kit (Bio-Rad) testing the CSF sample for the presence of soluble antigens of *Streptococcus pneumoniae, Haemophilus influenzae* type b*, Neisseria meningitidis* group A, group B/*E.coli* K1, group C, group Y/W135, and *Streptococcus* group B was negative. Microscopic examination showed several neutrophil granulocytes and a very few number of hardly dyed short rods or elongated cocci that seemed to be Gram-negatively stained at first examination. To exclude tuberculous meningitis Ziehl-Neelsen staining was performed, but no acid-fast bacilli were detected. At the 18–24-h and 48 h readings bacterial growth was negative both on the blood and on the chocolate culture media. Only the enrichment broth was slightly cloudy, indicating bacterial growth. On the next day, after 72 h of incubation tiny grey pinhead colonies were found on both blood and chocolate agar plates that were catalase and oxidase negative. Similar colonies were seen on the anaerobic plates. Irregular small Gram-positive bacilli and coccobacilli were observed in the microscope. The bacterium was identified as *A. turicensis* by VITEK 2 ANC ID Card (Biomerieux) and API Coryne (Biomerieux). The identification was verified by molecular method. PCR was performed with primers that amplified 1343 bp fragment of bacterial 16 s rRNA coding sequence [19]. PCR thermal profile was as follows: 5 min at 94 °C, 40 cycles of 94 °C for 1 min, 55 °C for 1 min and 72 °C for 1 min and a final extension at 72 °C for 2 min [20]. PCR amplicons have been purified by Qiagen PCR Purification Kit (Qiagen, GmbH, Hilden, Germany) and have been sequenced (BIOMI Kft., Gödöllő, Hungary). Analysis of nucleic acid sequence was done based on online tools of NCBI GenBank thus, the strain was identified as *A. turicensis*.

Antibiotic susceptibility of tested strain was performed by E-test after the European Committee on Antimicrobial Susceptibility Testing (EUCAST) recommendations. The isolated strain was susceptible to penicillin, ampicillin, imipenem, meropenem and vancomycin at concentrations ranging from 0.125 to 0. 5 mg/L.

As the patient’s condition did not improve a left side mastoid surgery was performed. During the mastoidectomy intraoperatively removed purulent discharge was sent to our Microbiology laboratory. After two days of incubation colonies grew on the agar plates that were similar to those isolated from the CSF sample. Identification both by biochemical reactions of automated systems and by 16S rRNA PCR and sequencing resulted *A. turicensis*. Moreover, the strain presented the same antibiotic MIC values as isolate of the CSF sample.

**Fig. 1:**
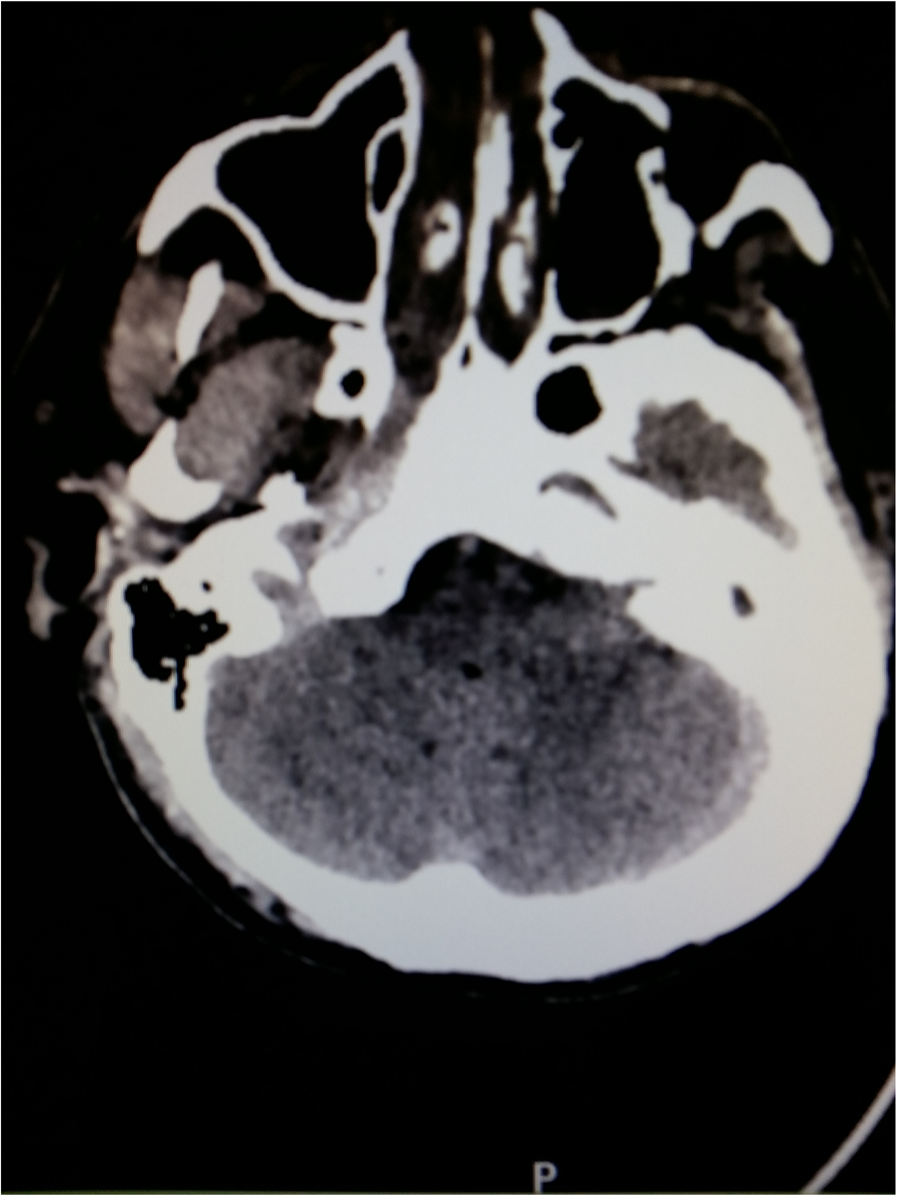
Skull CT: horizontal slice of brain with left side mastoiditis

**Fig. 2:**
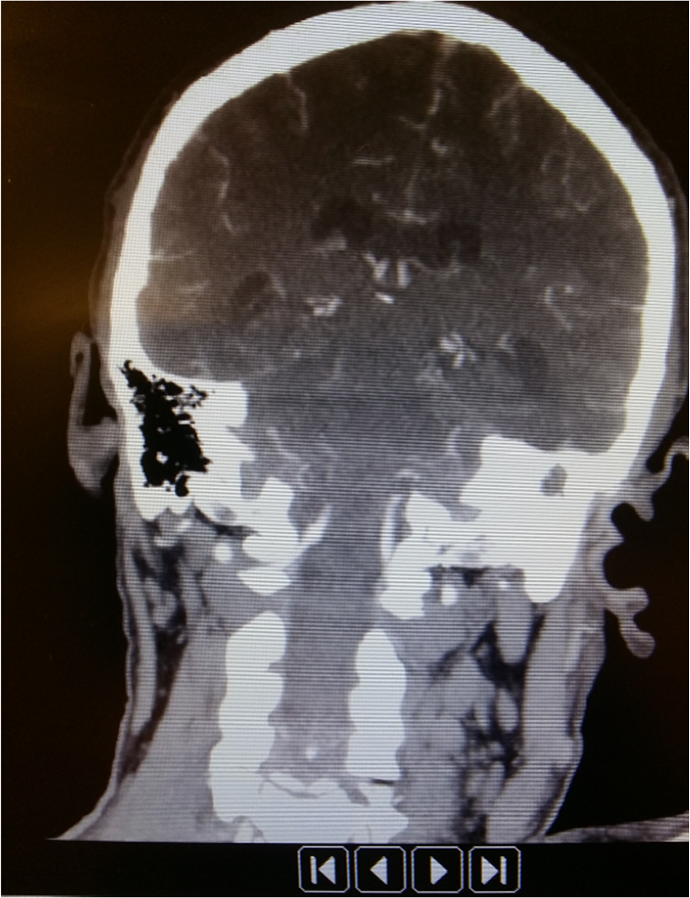
Skull CT: coronal slice of brain with left side mastoiditis

Postoperative bleeding occurred leading to the necessity of reoperation. On the third day of postoperative period severe polyuria was presented with dilated pupils insensible to light. The skull CT scan revealed cerebral herniation, intensifying oedema and sinus thrombosis. (Figures 1 and 2). Unfortunately, there was no chance of reoperation because the patient passed away.

After 5 days of aerobic and anaerobic incubation of the blood cultures no bacterial growth was detected. Autopsy was performed that confirmed the clinical diagnosis of cerebral herniation due to the purulent meningitis and the consequent oedema in the CNS. Moreover, a greenish-yellow discharge accumulated at the brain stem and in the lateral ventricules. During histopathology an infiltration of granulocytes at the meninges was seen and suspected signs of actinomycosis was also observed.

## Discussion and conclusions

*Actinomyces spp.* resides on mucous surfaces typically in the alimentary and genital tracts. On the other hand, *Actinomyces* spp. can rarely cause CNS infections due to the contiguous extension of neighbouring foci, such as paranasal sinusitis, or hematogenous seeding from a distant infected site such as a dental abscess, lungs and cervicofacial region although no such apparent foci are observed in one-third of cases. The typical manifestation of Actinomyces CNS infection is brain abscess, but Smego reported that 13% of CNS lesions of actinomycosis involve meningitis [17]. *A. turicensis* has been described as a potential pathogen mostly in genital and skin-related infections, however here we presented a case of CNS infection, namely *A. turicensis* meningitis as a complication of purulent mastoiditis with fatal outcome. In our patient an identical *A. turicensis* strain has been identified from a purulent mastoiditis and in meningitis, indicating the invasion of the pathogen from the infectious focus to CNS. There were no signs or symptoms of lung or skin actinomycosis. Anaerobes are found to be rare causative agents of meningitis but as our case report has shown it is worth to keep it in mind as possible pathogens of CNS. It may occur in patients living in poor socioeconomic conditions (e.g: homeless and alcohol abuse) and suffering from paranasal sinusitis or inflammation of other neighbouring foci. Here, we propose the need for anaerobic cultures of CSF specimens that is essential to identify *Actinomyces spp*. as well as other anaerob bacteria in CNS infections.

## Abbreviations

16S rRNA: 16S ribosomal subunit ribonucleic acid
CAMP test: Christie-Atkins-Munch-Petersen synergistic heamolysis test
CNS: central nervous system
CSF: cerebrospinal fluid
CT: computer tomograph
EUCAST: European Committee on Antimicrobial Susceptibility Testing
MIC: minimum inhibitory concentration
NCBI: National Center for Biotechnology Information
PCR: polymerase chain reaction
WBC: white blood cell count

## References

[1] Hawkey PM, Jewes LA (1985). How common is meningitis caused by anaerobic bacteria?. J Clin Microbiol.

[2] Könönen E, Wade WG (2015). Actinomyces and related organisms in human infections. Clin Microbiol Rev.

[3] Wüst J, Stubbs S, Weiss N, Funke G, Collins MD (1995). Assignment of*Actinomyces pyogenes*-like (CDC coryneform group E) bacteria to the genus *Actinomyces* as *Actinomyces radingae* sp. nov. and *ctinomyces turicensis*sp. Nov. Lett Appl Microbiol.

[4] Sabbe LJ, Van De Merwe D, Schouls L, Bergmans A, Vaneechoutte M, Vandamme P (1999). Clinical spectrum of infections due to the newly described *Actinomyces* species *A. turicensis*, *A. radingae*, and *A. europaeus*. J Clin Microbiol.

[5] Dewhirst FE, Chen T, Izard J, Paster BJ, Tanner ACR, Yu W-H (2010). The human oral microbiome. J Bacteriol.

[6] Sarkonen N, Könönen E, Summanen P, Kanervo A, Takala A, Jousimies-Somer H (2000). Oral colonization with *Actinomyces* species in infants by two years of age. J Dent Res.

[7] Hoyles L, Clear JA, McCartney AL (2013). Use of denaturing gradient gel electrophoresis to detect *Actinobacteria* associated with the human faecal microbiota. Anaerobe.

[8] El Aila NA, Tency I, Claeys G, Verstraelen H, Saerens B, Santiago GLDS (2009). Identification and genotyping of bacteria from paired vaginal and rectal samples from pregnant women indicates similarity between vaginal and rectal microflora. BMC Infect Dis.

[9] Nikolaitchouk N, Hoyles L, Falsen E, Grainger JM, Collins MD (2000). Characterization of *Actinomyces* isolates from samples from the human urogenital tract: description of *Actinomyces urogenitalis* sp. nov. Int J Syst Evol Microbiol.

[10] Lu H, Qian G, Ren Z, Zhang C, Zhang H, Xu W (2015). Alterations of *Bacteroides sp.*, *Neisseria sp., Actinomyces sp.*, and *Streptococcus sp*. populations in the oropharyngeal microbiome are associated with liver cirrhosis and pneumonia. BMC Infect Dis.

[11] Smego RA, Foglia G (1998). Actinomycosis. Clin Infect Dis.

[12] Wong VK, Turmezei TD, Weston VC (2011). Actinomycosis. BMJ.

[13] Sama CB, Mbarga NF, Oben CE, Mbarga JA, Nfor EK, Angwafo Iii FF (2016). Massive paediatric cervicofacial actinomycoses masquerading as an ulcerative malignancy. BMC Infect Dis.

[14] Endo S, Mishima E, Takeuchi Y, Ohi T, Ishida M, Yanai M (2015). Periodontitis-associated septic pulmonary embolism caused by Actinomyces species identified by anaerobic culture of bronchoalveolar lavage fluid: a case report. BMC Infect Dis.

[15] Chotmongkol V, Panthavasit J, Chuesakoolvanich K (2002). Actinomycotic meningitis: report of a case. J Med Assoc Thail.

[16] Imamura K, Kamitani H, Nakayasu H, Asai Y, Nakashima K (2011). Purulent meningitis caused by Actinomyces successfully treated with rifampicin: a case report. Intern Med.

[17] Smego RA (1987). Actinomycosis of the central nervous system. Rev Infect Dis.

[18] Koda Y, Seto Y, Takeichi S, Kimura H (2003). Fatal subarachnoid haemorrhage complicating actinomycotic meningitis. Forensic Sci Int.

[19] Woo PCY, Cheung EYL, Leung K, Yuen K (2001). Identification by 16S ribosomal RNA gene sequenceing of an Enterobacteriaceae species with ambiguous biochemical profile from renal transplant recipient. Diagn Microbiol Infect Dis.

[20] Jenkins C, Ling CL, Ciesielczuk HL, Lockwood J, Hopkins S, McHugh TD (2012). Detection and identification of bacteria in clinical samples by 16S rRNA gene sequencing: comparison of two different approaches in clinical practice. J Med Microbiol.

